# Comparison of short-term clinical outcomes between robot-assisted and freehand pedicle screw placement in spine surgery: a meta-analysis and systematic review

**DOI:** 10.1186/s13018-023-03774-w

**Published:** 2023-05-16

**Authors:** Yiyang Li, Yan Wang, Xinlong Ma, Jianxiong Ma, Benchao Dong, Peichuan Yang, Yadi Sun, Liyun Zhou, Jiahui Shen

**Affiliations:** 1 Tianjin Hospital of Tianjin University (Tianjin Hospital), Tianjin, 300211 People’s Republic of China; 2 Tianjin Orthopedic Institute, Tianjin, 300050 People’s Republic of China; 3 Tianjin Key Laboratory of Orthopedic Biomechanics and Medical Engineering, Tianjin, 300050 People’s Republic of China

**Keywords:** Robotic surgery, Pedicle screw, Spine surgery, Short-term clinical outcomes

## Abstract

**Study design:**

Meta-analysis and systematic review.

**Background:**

Robot-assisted pedicle screw placement technique offers greater accuracy than the traditional freehand screw placement technique. However, it is controversial whether there is a difference between the two procedures in terms of improved clinical outcomes.

**Materials and methods:**

We systematically searched PubMed, EMBASE, Cochrane, and Web of Science to identify potentially eligible articles. Indispensable data such as the year of publication, study type, age, number of patients, sex distribution, and outcomes were extracted. The outcome indicators of interest included Oswestry disability index (ODI), visual analog scale (VAS) score, operative time, intraoperative blood loss, and post-operative length of stay. RevMan 5.4.1 was used for the meta-analysis.

**Results:**

A total of eight studies with 508 participants were included. Eight were related to ΔVAS, six were related to ΔODI, seven were related to operative time, five were related to intraoperative blood loss, and seven were related to the length of hospitalization. The results showed that, in terms of ΔVAS (95% CI, −1.20 to −0.36, *P* = 0.0003) and ΔODI (95% CI, −2.50 to −0.48, *P* = 0.004), robot-assisted pedicle screw placement technique scored higher than traditional freehand technique. Additionally, the intraoperative blood loss (95% CI, −140.34 to −10.94, *P* = 0.02) and the length of hospitalization (95% CI, −2.59 to −0.31, *P* = 0.01) for patients who underwent robotic-assisted pedicle screw placement were less than that of those who underwent the conventional freehand screw placement. No significant difference was found between robot-assisted techniques and conventional freehand techniques in pedicle screw placement in surgical time (95% CI, −2.24 to 26.32, *P* = 0.10).

**Conclusions:**

Robot-assisted technique helps improve short-term clinical outcomes, reduce intraoperative blood loss and patient suffering, and shorten recovery time compared to the freehand technique.

## Introduction

Pedicle screw fixation is an effective treatment option for all spinal disorders. Pedicle screw placement provides an excellent three-column fixation and is widely used in spinal surgery. However, the traditional freehand screw placement method has limitations because the operator's field of vision and body posture are limited by space, which affects the accuracy of screw placement and can lead to pedicle violations. The misplacement rate of conventional freehand pedicle screws ranges from 5 to 41% in the lumbar spine and 3% to 55% in the thoracic spine [1], which may lead to nerve injury, dural tearing, and other complications. In recent years, the development of robot-assisted pedicle screw placement has gradually advanced, and an increasing number of clinical cases have shown the advantages of robot-assisted screw placement over freehand screw placement in terms of improved accuracy and reduced intraoperative bleeding [2]. Feng et al. [3] performed a randomized controlled trial (RCT). They reported that 98.5% of robot-assisted pedicle screw placements achieved an accuracy of grade A. Fu et al. performed a meta-analysis [2] and reported that robot-assisted techniques were more accurate in pedicle screw placement than freehand techniques.

However, whether robot-assisted techniques are superior to freehand techniques in terms of post-operative clinical outcomes remains unclear. Only a few reviews and meta-analyses have focused on this issue. Karamian et al. [4] concluded that the benefits of robot-assisted pedicle screw placement may not apply to patients in terms of clinical outcomes, in a study that compared the post-operative clinical outcomes of robot-assisted screw placement with freehand screw placement. In contrast, Cui et al. [5] reported that the robot-assisted technique significantly reduced post-operative suffering compared with freehand screw placement, in a retrospective cohort study. Therefore, whether robot-assisted pedicle screw placement can improve clinical outcomes remains controversial. Thus, this meta-analysis aimed to determine whether robot-assisted pedicle screw placement technique offers an advantage in short-term clinical outcomes compared with the freehand screw placement technique. The clinical indicators we used were the difference between the pre- and post-operative visual analog scores (ΔVAS), difference between the pre- and post-operative Oswestry disability index (ΔODI), operative time, intraoperative blood loss, and the length of hospital stay (post-operative stay).

## Materials and methods

### Data search strategy

This meta-analysis followed the Preferred Reporting Items for Systematic Reviews and Meta-Analysis procedures. We systematically searched the PubMed, EMBASE, Cochrane, and Web of Science databases to identify potentially eligible articles. All the databases were updated on November 10, 2022. Medical subject headings (MeSHs) and free-text words were used to search for potential literature. The PubMed, EMBASE, Cochrane, and Web of Science databases were searched using the following keywords: ‘‘robotics’’ (or ‘‘robot’’ or ‘‘robotic’’ or ‘‘robotics’’) (MeSH) and ‘‘pedicle screws’’ (or ‘‘bone screws’’ or ‘‘spine’’ or ‘‘spinal column’’) (MeSH) (Table 1). Two reviewers (YYL and YDS) independently searched all titles and abstracts, and the references of relevant studies were reviewed for additional relevant literature. Any discrepancies were resolved through discussion or consultation with a third reviewer (WY).

**Table 1:** Search strategy for each database

Database	Search strategy
Pubmed	("Robotics"[MeSH Terms] OR "robot"[All Fields] OR "robotics"[All Fields] OR" robotic"[All Fields]) AND ("pedicle screws"[MeSH Terms] OR "pedicle"[All Fields] OR "screws"[All Fields] OR "pedicle screws"[All Fields] OR "bone screws"[MeSH Terms] OR "bone screw" [All Fields] OR "traditional trajectory screw"[All Fields] OR "spine"[MeSH Terms] OR "spine"[All Fields] OR "Vertebral Column"[All Fields] OR "Vertebral Columns"[All Fields] OR "Spinal Column"[All Fields] OR "Spinal Columns"[All Fields] OR "Vertebra"[All Fields] OR "Vertebrae"[All Fields])
Embase	#1: "pedicle screws" OR "Pedicle Screw" OR "Zygapophyseal Joint" OR "Zygapophyseal Joints" OR "Facet Joint" OR "Facet Joints" OR "Vertebral Column" OR "Vertebral Columns" OR "Spinal Column" OR "Spinal Columns" OR "Spine" OR "Vertebra" OR "Vertebrae" OR "bone screws" #2: "Robotics" OR "robot" OR "robotics" OR" robotic" #3 #1 AND #2
Cochrane library	#1 "pedicle screws" OR "Pedicle Screw" OR "Vertebral Column" OR "Vertebral Columns" OR "Spinal Column" OR "Spinal Columns" OR "Spine" OR "Vertebra" OR "Vertebrae" #2 "robot" OR "robotics" OR "robotic" #3 #1 AND #2
Web of Science	#1 pedicle screws OR Pedicle Screw OR Vertebral Column OR Vertebral Columns OR Spinal Column OR Spinal Columns OR Spine OR Vertebra OR Vertebrae #2 robot OR robotics OR robotic #3 #1 AND #2

### Study selection

The following inclusion criteria were identified before the search: articles involving spinal robotic pedicle screw placement, articles with post-operative computed tomography scans to assess accuracy, and articles providing sufficient data for meaningful comparison (more than 10 pedicle screws per study group). The exclusion criteria were as follows: duplicate publications; articles without traditional freehand pedicle screw placement in the control group; and articles that did not include patient visual analog scores, Oswestry disability index, and post-operative length of stay. Both RCTs and retrospective cohort studies (RCSs) were eligible for inclusion. Only human studies were considered. The inclusion of studies was not limited by sample size or publication type. Review articles and commentaries were excluded from the analysis.

### Quality assessment and data extraction

The two reviewers independently assessed all included studies according to the Cochrane risk-of-bias criteria and the Newcastle–Ottawa Quality Assessment Scale using the risk-of-bias tool. The Cochrane risk-of-bias criteria were used to assess the quality of RCTs in terms of selection, performance, detection, attrition, reporting, and other biases. We defined other biases as differences in baseline characteristics between the experimental and control groups. Retrospective cohort studies were evaluated using the Newcastle–Ottawa Quality Assessment Scale, rated from 0 to 9 stars. Six stars or higher indicated sufficiently high quality.

The two reviewers independently performed data extraction. Disagreements were resolved through discussion or consultation with the third reviewer. Indispensable data such as the year of publication, study type, age, number of patients, sex distribution, and outcomes were extracted. The outcome indicators of interest included ODI, VAS score, operative time, intraoperative blood loss, and post-operative length of stay.

## Results

### Characteristics of the included studies

**Fig. 1:**
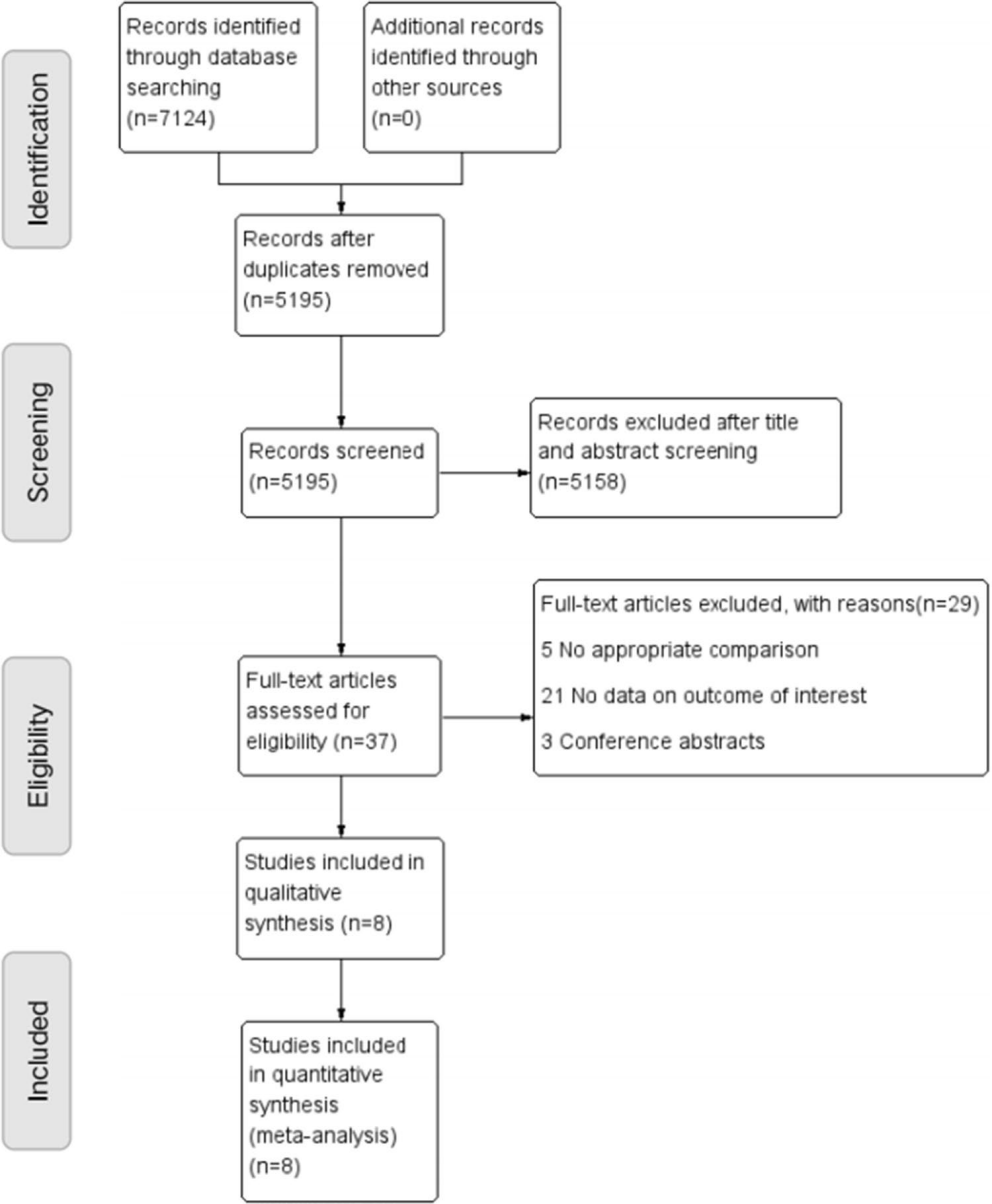
Schematic diagram of the study procedure

**Fig. 2:**
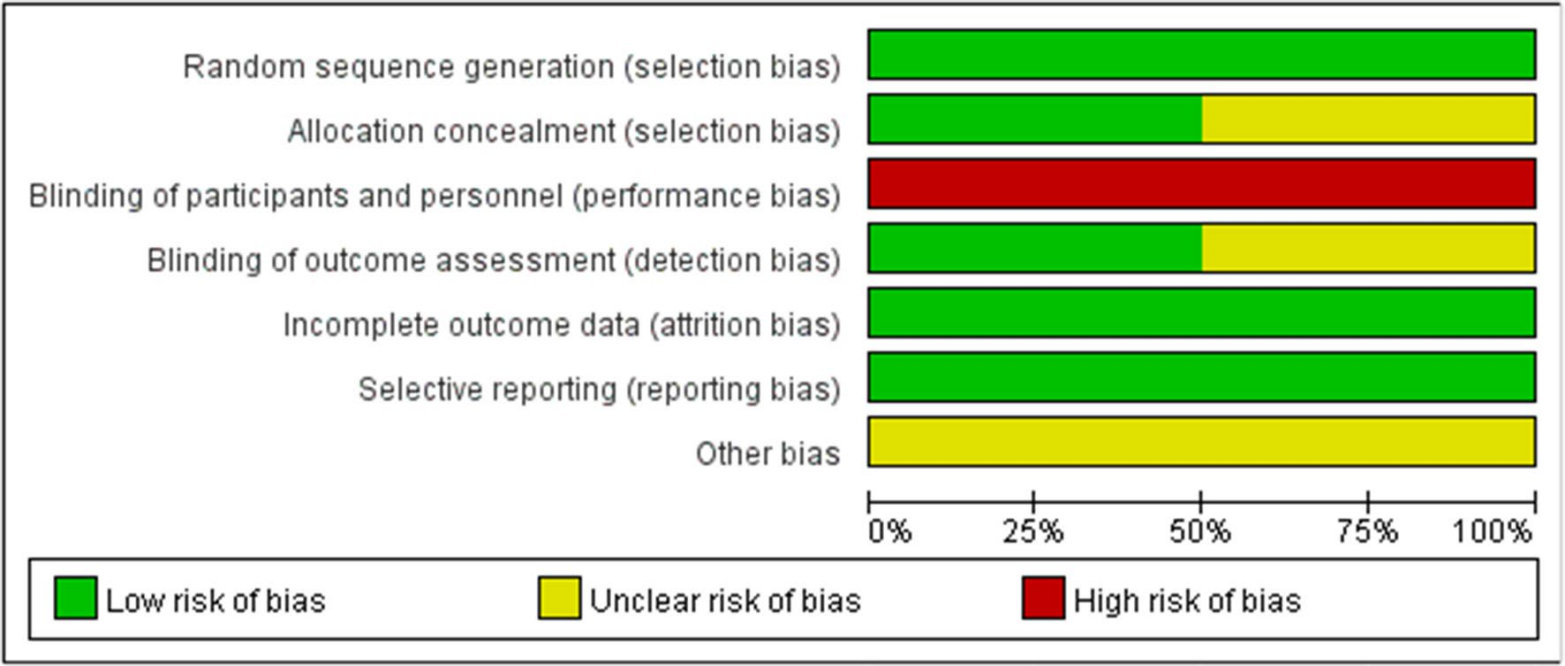
Summary of the risk of bias of the included randomized controlled trials

The process of inclusion of these studies is illustrated in Fig. 1. A total of 7124 relevant studies were identified through the web search. A total of 1929 studies were excluded, because they were duplicates. After assessing the titles and abstracts, 5158 studies were excluded because their contents did not meet the criteria. After verifying the full text of the remaining 37 studies, four RCTs [6–9] and four RCSs [5, 10–12] with a total of 508 patients were finally included in this meta-analysis. The main characteristics of the included studies are summarized in Table 2. Baseline information was balanced and comparable across the eight studies. Among the eight studies, the type of robot used in two studies was Renaissance and in six studies was TiRobot. RCTs were shown to have a low risk of bias (Fig. 2 and Table 3), and all RCSs had evaluation scores greater than six stars (Table 4). All included studies demonstrated satisfactory quality.

**Table 2:** Characteristics of the included studies

Characteristics of included studies									
Study	Study type	Age, y		No. of patients	Sex (M/F)		Robot type	No. of A-level Screws/All Screws	Quality assessment/nos score
		RA	FH		RA	FH			
Feng [3]	RCT	63.45 ± 4.56	64.22 ± 6.19	*N* = 80 RA = 40 FH = 40	16/24	15/25	TiRobot	RA:167/170 FH:162/174	RCT
Cui [6]	RCT	51.3 ± 9.8	54.1 ± 10.2	*N* = 48 RA = 23 FH = 25	4/19	6/19	TiRobot	RA:87/92 FH:85/100	RCT
Wang [9]	RCT	57.46 ± 8.68	57.69 ± 9.15	*N* = 123 RA = 61 FH = 62	16/45	21/41	TiRobot	RA:234/274 FH:196/282	RCT
Hyun [7]	RCT	66.5 ± 8.1	66.8 ± 8.9	*N* = 60 RA = 30 FH = 30	9/21	8/22	Renaissance	RA:127/130 FH:133/140	RCT
Cui [5]	RCS	52.5 ± 9.3	54.1 ± 10.2	*N* = 41 RA = 16 FH = 25	1/15	6/19	TiRobot	RA:61/64 FH:85/100	⭐⭐⭐⭐⭐⭐⭐
Lin [11]	RCS	NA	NA	*N* = 52 RA = 24 FH = 28	11/13	13/15	TiRobot	RA:124/132 FH:145/158	⭐⭐⭐⭐⭐⭐⭐⭐
Tian [12]	RCS	NA	NA	*N* = 58 RA = 28 FH = 30	18/10	19/11	Renaissance	RA:150/160 FH:144/170	⭐⭐⭐⭐⭐⭐⭐⭐
Zhang [10]	RCS	40.23 ± 12.19	42.88 ± 10.31	N = 46 RA = 22 FH = 24	12/10	15/9	TiRobot	RA:120/128 FH:107/134	⭐⭐⭐⭐⭐⭐⭐

*FH* freehand group, *NOS* Newcastle–Ottawa Scale, *RA* robot-assisted group, *RCT* randomized controlled trial, *RCS* retrospective cohort study

**Table 3:** Risk of bias assessment of the randomized controlled trials

RCT	Random sequence generation	Allocation concealment	Blinding of participants and personnel	Blinding of outcome assessment	Incomplete outcome data	Selective reporting	Other bias
Feng et al	Low risk	Unclear risk	High risk	Low risk	Low risk	Low risk	Unclear risk
Cui et al	Low risk	Low risk	High risk	Low risk	Low risk	Low risk	Unclear risk
Wang et al	Low risk	Unclear risk	High risk	Unclear risk	Low risk	Low risk	Unclear risk
Hyun et al	Low risk	Low risk	High risk	Unclear risk	Low risk	Low risk	Unclear risk

**Table 4:** Risk of bias assessment of the cohort studies

RCS	Selection	Comparability	Outcome	Total
Cui et al	**⭐⭐⭐**	**⭐**	**⭐⭐⭐**	**⭐⭐⭐⭐⭐⭐⭐**
Lin et al	**⭐⭐⭐⭐**	**⭐**	**⭐⭐⭐**	**⭐⭐⭐⭐⭐⭐⭐⭐**
Tian et al	**⭐⭐⭐⭐**	**⭐**	**⭐⭐⭐**	**⭐⭐⭐⭐⭐⭐⭐⭐**
Zhang et al	**⭐⭐⭐**	**⭐**	**⭐⭐⭐**	**⭐⭐⭐⭐⭐⭐⭐**

### ΔVAS and ΔODI

**Fig. 3:**
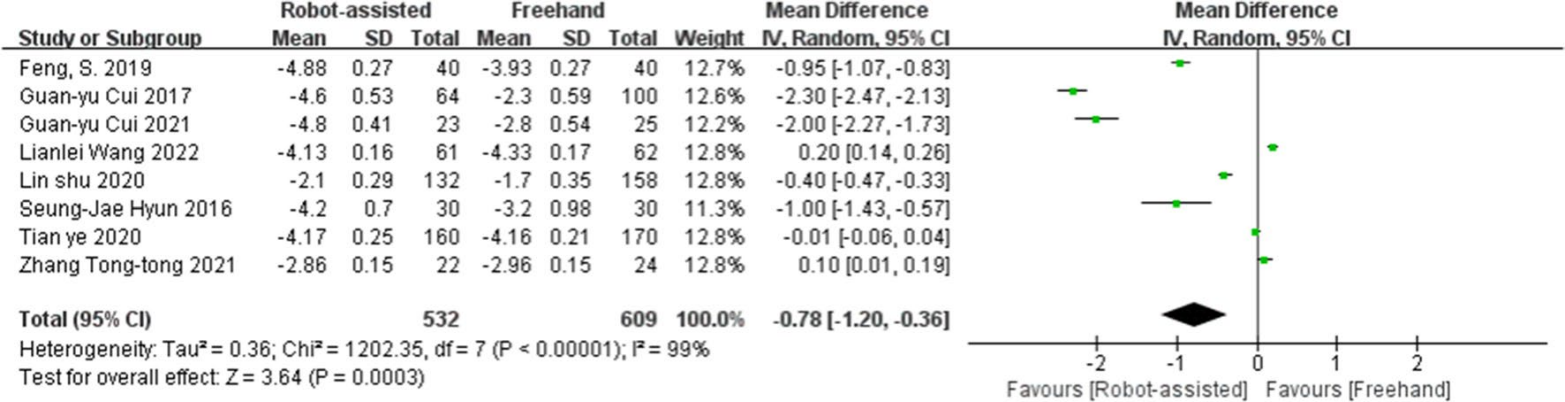
Forest plot of ΔVAS for robot-assisted technique versus the conventional freehand technique of pedicle screw placement

**Fig. 4:**
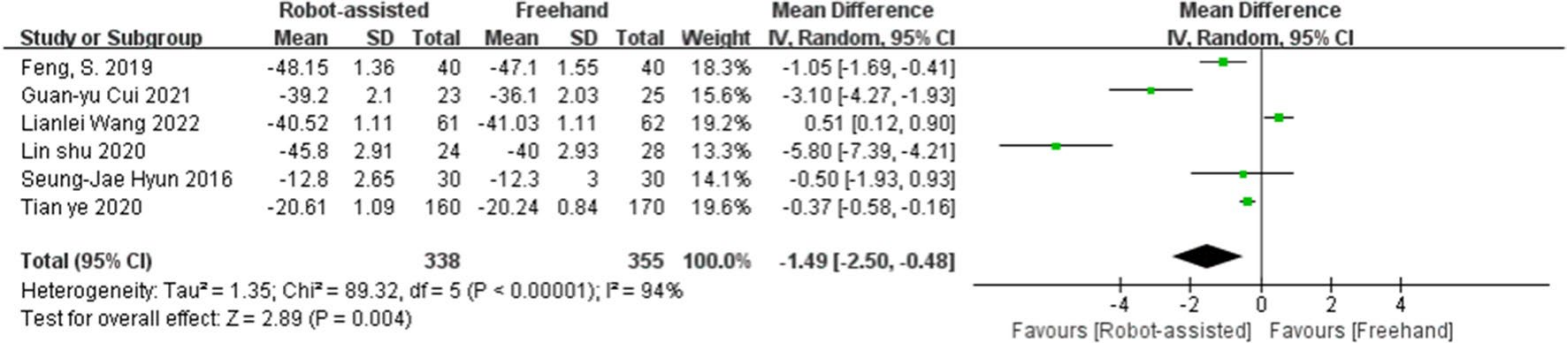
Forest plot of ΔODI for robot-assisted technique versus the conventional freehand technique of pedicle screw placement

Eight studies provided data on pre- and post-operative VAS scores; seven studies provided data on pre- and post-operative ODIs. After obtaining the data from each study, we calculated the ΔVAS and ΔODI for each study, using statistical methods. The results showed that in terms of ΔVAS (SMD = −0.78, 95% CI, −1.20 to −0.36, *P* = 0.0003; Fig. 3) and ΔODI (SMD = −1.49, 95% CI, −2.50 to −0.48, *P* = 0.004; Fig. 4), robot-assisted pedicle screw placement technique scored higher than the traditional freehand technique.

### Operative time

**Fig. 5:**
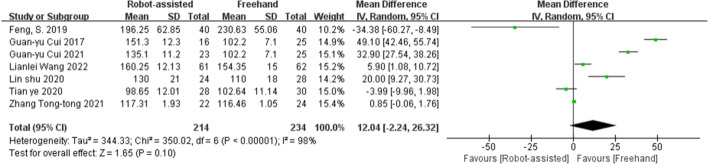
Forest plot of operative time for robot-assisted technique versus the conventional freehand technique of pedicle screw placement

Seven studies provided data on operative time. No significant difference was found between robot-assisted techniques and conventional freehand techniques in pedicle screw placement in surgical time (SMD = 12.04, 95% CI, −2.24 to 26.32, *P* = 0.10; Fig. 5).

### Intraoperative blood loss

**Fig. 6:**
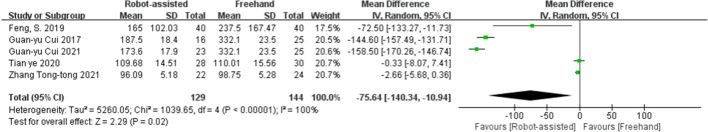
Forest plot of intraoperative blood loss for robot-assisted technique versus the conventional freehand technique of pedicle screw placement

Five studies provided data on intraoperative blood loss. The results showed that the intraoperative blood loss for patients who underwent robotic-assisted pedicle screw placement (SMD = −75.64, 95% CI, −140.34 to −10.94, *P* = 0.02; Fig. 6) was less than that those who underwent the conventional freehand screw placement.

### Length of hospitalization

**Fig. 7:**
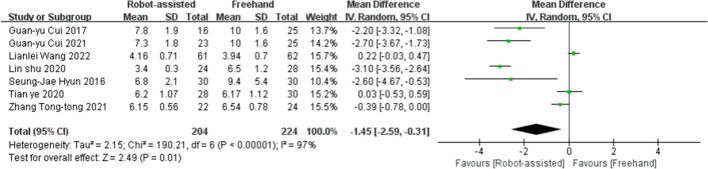
Forest plot of for post-operative stay for robot-assisted technique versus the conventional freehand technique of pedicle screw placement

Seven studies provided data on the length of hospitalization. The results showed that the post-operative length of hospitalization for patients who underwent robotic-assisted pedicle screw placement (SMD = −1.45, 95% CI, −2.59 to −0.31, *P* = 0.01; Fig. 7) was less than that those who underwent the conventional freehand screw placement.

## Discussion

Robot-assisted pedicle screw placement has been designed to improve the accuracy and safety of pedicle screw placement. Theoretically, robot-assisted screw placement technology has the advantages of high precision, repeatability, and fatigue resistance compared with traditional freehand screw placement technology. A meta-analysis [13] was conducted to demonstrate the benefits of the robot-assisted technology in terms of screw placement accuracy. However, the benefits of robot-assisted technology remain controversial. Regarding post-operative rehabilitation outcomes, most available meta-analyses used direct comparisons of post-operative VAS scores and ODIs. They concluded that there was no significant difference in terms of clinical outcomes between robot-assisted and conventional freehand screw placement techniques [14]. However, we believe that their comparisons have certain limitations. In clinical studies addressing post-operative rehabilitation outcomes, the initial VAS scores and ODIs differed between patients in the experimental and control groups, and experimental conclusions obtained can be inaccurate if only the corresponding post-operative scores were compared without considering the differences in their pre-operative scores; therefore, statistical methods were used to address this issue.

In this meta-analysis, eight studies with a total of 2408 screws were included to compare the differences between robot-assisted and traditional freehand pedicle screw placement methods in terms of five clinical indicators: ΔVAS, ΔODI, operative time, intraoperative blood loss, and length of hospitalization. The results for four metrics were significantly different between the two methods. The robot-assisted technique was superior to the traditional freehand screw placement technique in four metrics. Hence, the results recommend the use of the robot-assisted technique for screw placement.

Notably, we compared the difference between the pre- and post-operative VAS scores and ODIs of patients who underwent the two procedures. Because the VAS scores and ODIs gradually decrease with time, as reported in previous studies, there is no significant difference in long-term clinical outcomes between the two surgical techniques [15]. It is equally important to focus on short-term clinical outcomes in the clinical practice of healthcare professionals. A good short-term clinical outcome means that patients recover faster, experience less pain, and incur less expense from rehabilitation and hospitalization; therefore, we uniquely compared and analyzed the short-term clinical outcomes of the two surgical modalities.

The original data of each clinical study were not available; therefore, we used statistical methods to calculate the mean and standard deviation of ΔVAS and ΔODI in each study. The calculation of VAS score is presented as an example of a specific calculation method.

The pre-operative visual analog score was set as $VAS_{1}$, and its mean was $\mu \left( {VAS_{1} } \right)$, with a standard deviation of $\sigma \left( {VAS_{1} } \right)$. The post-operative visual analog score was $VAS_{2}$, and its mean was $\mu \left( {VAS_{2} } \right)$, with a standard deviation of $\sigma \left( {VAS_{2} } \right)$. When the index used to measure the reduction in the VAS score was ${\Delta }VAS$ with mean $\mu \left( {{\Delta }VAS} \right)$ and standard deviation $\sigma \left( {{\Delta }VAS} \right)$, and the number of patient cases was $n$, then:

$$\begin{aligned} \mu \left( {\Delta VAS} \right) & = \mu \left( {VAS_{1} } \right) - \mu \left( {VAS_{2} } \right) \\ \sigma \left( {\Delta VAS} \right) & = \sqrt {\sigma^{2} \left( {VAS_{1} } \right)/n + \sigma^{2} \left( {VAS_{2} } \right)/n} \\ \end{aligned}$$

In previous studies, patients were followed up over time to investigate the advantages of robot-assisted screw placement technique over the traditional freehand screw placement technique in terms of clinical outcomes. The follow-up results were compared directly. Su et al. [16] and Lee et al. [17] compared the ODIs and VAS scores of patients after 1 or 2 years and concluded that there was no significant difference between the two methods in terms of long-term clinical outcomes. However, with technological advances in the development of robot-assisted pedicle screw placement technique, controversy regarding its accuracy has been resolved to some extent. An RCT by Kim et al. [18] and a meta-analysis by Lee et al. [19] showed that the robot-assisted screw placement has a significantly higher accuracy than that of the traditional freehand technique and has a lower rate of proximal tuberosity joint invasion. In this context, there is a need to re-evaluate the advantages of robot-assisted pedicle screw placement technique in post-operative rehabilitation; however, the available meta-analyses do not yet include an analysis of short-term clinical outcomes. In this study, we compared the short-term clinical outcomes of the two surgical methods using the difference between the pre- and post-operative VAS scores and ODIs as a measure of short-term post-operative clinical outcomes. We also compared the operative time, the intraoperative blood loss, and the post-operative length of hospitalization.

We believe that the ultimate goal of developing orthopedic surgical robots is to reduce patient suffering and that patients' subjective perceptions of post-operative recovery outcomes are an important measure of the success of robot-assisted technology. This study identified significant differences between the robot-assisted and freehand screw placement techniques as indicators of short-term post-operative clinical outcomes. The results showed that the robot-assisted screw placement technique was more effective in reducing the patient's VAS score, ODI, and intraoperative blood loss than the freehand screw placement technique and thus reduced the patient's post-operative hospital stay. Furthermore, the robot-assisted screw placement technique did not significantly prolong the operative time.

The present study has some limitations. First, four of the included studies were retrospective cohort studies, and the level of evidence was not as high as that of RCTs. Second, only two robot models were included in this study, and six of the eight included studies used the same robot model, which may have rendered the conclusions of this study inapplicable to other robot models. Despite these limitations, all RCSs included in this meta-analysis had scores ≥ 7 stars and were of high quality.

## Conclusion

The robot-assisted pedicle screw placement technique showed a significant reduction in the patients’ VAS scores, ODIs, intraoperative blood loss, and post-operative length of hospitalization compared with the traditional unassisted technique. And the robot-assisted screw placement technique did not significantly prolong the operative time. These results suggest that robot-assisted techniques help improve short-term clinical outcomes, reduce patient suffering, and shorten recovery time compared with the freehand technique.

## Data Availability

The datasets used and/or analyzed during the current study are available from the corresponding author on reasonable request.

## Abbreviations

VAS: Visual analog scale
ΔVAS: Difference between pre-operative and post-operative VAS values
ODI: Oswestry disability index
ΔODI: Difference between pre-operative and post-operative ODI values
NOS: Newcastle–Ottawa scale
MD: Mean difference
CI: Confidence interval
OR: Odds ratio
RCT: Randomized controlled trial
RCS: Retrospective cohort study
